# The General Hospital at Birmingham

**Published:** 1889-10-26

**Authors:** 


### THE HOSPITAL WORKSHOP.

### No. XII.—THE GENERAL HOSPITAL AT BIRMINGHAM.

(BY OUR SPECIAL COMMISSIONER).

The board-room of this institution contains some good busts and paintings. Among the latter is a portrait by Sir Joshua Reynolds of John Ash, founder and first physician. It is a bold example of the artist's work, and its rich colouring is fresh as the day it left the easel. Rumour says that Government has made a large bid for this magnificent canvas, with a view to placing it in the National Gallery, and few people in this romantic age would murmur if the governors were to sell the original and place a copy on their walls. The proceeds would no doubt be devoted by these stern economists towards paying off the steadily increasing debt with which the public is yearly confronted. The date of the picture—1779—also marks the opening of the hospital, which, at the time of its foundation, was in a comparatively open position, and the name of Summer-lane, where it stands, was not altogether inappropriate. There is still a clear space round the building, but the rapid development of Birmingham has completely hemmed in and environed the hospital with various arrangements of bricks and mortar, and with other undesirable factors of town life. Hard by is a sluggish canal, with a coal yard on its nether bank, and in the immediate vicinity are works of various kinds, notably one for making sulphuric acid, the fumes of which often pervade the whole neighbourhood. Add to all this a number of tall chimneys that seem to be always "stoking up," and the scene quite warrants the bequest of Miss Rylands leaving £25,000 to the committee on condition that the site of the hospital be changed within five years. Such a course, however, would involve a large additional expenditure; for instance, the ground alone in a central position would cost something like £70,000. On the other hand, judging from the reports, results of cases seem fairly good, and the spot is within easy reach of places where accidents are most likely to occur. It has been rumoured from time to time that the sanitary arrangements are old and imperfect, and enquiry elicited the fact that inmates occasionally suffer from epidemics of sore throat and other illness commonly associated with faulty hygiene.

Admission is free to accidents and urgent cases, otherwise a subscriber's ticket is required. During 1888, 678 in-patients were admitted by tickets and recommendations, while 2,662 were received without any such qualification. The total number relieved for the same year reached 47,690. Since the first foundation 1,404,521 persons have partaken of the benefits of the institution. The pay system is not in force, but, on the other hand, there is no attempt at systematic enquiry into patients' means, and it is to be feared that abuses are not infrequent. It is said, for instance, that applicants are able to purchase tickets outside at a certain market value, an out-patient note being worth half-a-crown. Whether this be true or not, any man presenting himself with a ticket may, under existing arrangements, demand to be taken in for six weeks, that is to say, if his state of health warrant such admission. Some time ago the Hospital Saturday Fund were instrumental in having the words "object of charity" struck off the tickets and "object of relief" substituted. So that in the latter capacity those who have tickets demand admission as a right. Then, again, many employers exact a penny a week from their men, expending the sum thus collected in buying tickets, and those who use them, naturally enough, regard the hospital in the light of a provident institution whose services they have bought and paid for. Public interest is now being roused all over the country in matters relating to charitable institutions, and such facts as these are not likely to be overlooked. Nor can the awakening feeling of the medical profession on these points be disregarded by hospital authorities. The appointment of a royal commission of enquiry must, sooner or later, follow the present agitation.

Under the guidance of the resident officers, your commissioner made the round of this extensive building, and a few of the chief points of interest may be briefly narrated.

The main door opens into a square hall that boasts a handsome tessellated pavement. The ceiling is low, and the walls are panelled with boards that from their funereal look might well be the mourning hatchments of departed donors. A passage right and left leads to the surgery and other offices usually clustered near the entrance of a hospital. The wards are plain and comfortable, ornamented with a few flowers and pictures, and floored with polished oak. Their shape is very variable, from the fact that the building, like some old-fashioned straggling country houses, has grown to its present size by many additions. The iron bedsteads are of a curious shape with four tall posts and an over-arching framework. This somewhat ancient pattern is useful for curtains, but must be inconvenient for any cases that require dressing or manipulation.

Here is a patient between forty and fifty who has had a "stroke." He was picked up unconscious in the street and brought here by the police. That was nearly a month ago, and he has not recovered his reason sufficiently to recognise his friends, although he answers questions in a confused kind of way. In Birmingham many of the constables are certificated ambulance men, and the practical good of their training is being constantly put to the proof. St. John's classes on the subject carefully teach how to distinguish between drunkenness and fits, and were such knowledge more widely diffused those painful cases would be avoided where a dying man is thrust into a police cell under the impression that he is drunk.

In another medical ward was a man of forty with heart disease that had existed since infancy. He was propped up in bed, and blue and breathless from impeded circulation. Before an infant breathes, the blood, instead of being pumped through the lungs, passes from one side of the heart to the other by an opening which closes soon after birth. In some rare cases the communication persists, and a portion of the blood only is sent through the lungs. Persons thus affected do not as a rule live very long, and it is quite exceptional for one to reach the age of this patient. Some evolutionists regard this imperfection as a trace of some ancestral condition. In the reptiles, the two sides of the heart communicate, and the body is supplied with mixed blood.

Many persons come to this hospital poisoned by various metals, such as lead, copper, or brass, used in their work. Lead poisoning is probably of much commoner occurrence than is suspected. Here is a girl of twenty-three, who shows well-marked symptoms. She worked in a paper-bag factory for six years. Her first signs were obstinate dyspepsia and severe colic pains; at the same time she became pale, bloodless, and weak. A blue line formed along the gums, at the margins of the teeth, but the common symptoms of headache and a sweet taste in the mouth appear to have been absent. She had rheumatic pains round the joints. (Obstinate rheumatism and gout, with secondary kidney disease, constantly follow lead poisoning.) Her eyes were unaffected; but the muscles at the back of the forearms are wasted and paralysed, causing "wrist-drop," in which condition the hands droop powerlessly when the arms are stretched out palms downwards. This palsy of muscles is one of the later and more serious stages of the disease; others are loss of sight, epilepsy, slowed heart, and various paralyses. Lead poisoning is now attracting a good deal of attention, and is sometimes contracted from the drinking water that has passed through lead pipes or cisterns. The frequency of its occurrence among workpeople is almost beyond belief, and an enquiry is urgently needed into this subject with a view to legislation. Painters are commonly affected—everyone knows how they are nearly always pale-faced and martyrs to rheumatism and gout. That is because they are poisoned by the white lead that is used in nearly all paints. Lead is the basis of many pigments, and in that form has most likely caused the illness of the girl before us, for she had to make her bags from differently coloured papers. It is a curious fact that artists, who often eat hurried meals in their studios, manage to escape "plumbism," as it is called.

Away from the main building are two wards, holding about 30 patients, for burns and occasionally a few other cases. The local Kyrle Society has done a great deal here in the way of decoration. Round the top of one ward runs a large dado, showing a number of bare and leafless tree-tops, in which are owls, jays, magpies, and other birds. Contrary to what might be expected, the bulk of the burns do not come from the factories. Now and then the Ammunition Works send in some explosion burns, or men are scalded at the boilers. The greater number of cases, however, are children who have met with accidents. They have been left at home alone, with no guard to the fire, and the neighbours often say they found the children in flames.

In this ward is a boy of four, who has been knocked down and seriously injured by the engine of a steam tram. Besides much bruising about the body, both legs and the left thigh were broken, the hip was injured, and the head severely damaged, but no fracture could be made out. He was brought here a fortnight ago and has done wonderfully well, for he can now tell his name and says he would like to go home. Accidents of this kind are very fatal and far too common. In front of the engine there is a board, called a "protector," which can be shot out by the driver, and is intended to throw people who are run down clear of the lines. Within a week of the date of writing this article two cases have been reported in the papers. In the first a boy was saved by the prompt action of the "protector," but in the other there was no time to put it in action, as the unfortunate child ran out of an alley straight under the tram.

The nurses' home is a convenient house away from the hospital. It consists of a central hall with top skylight and tiers of bedrooms opening on to galleries. In addition there are recreation and bath-rooms, but the dining-hall is in the main building.

This institution contains an immense amount of material for the study of different accidents and diseases, and their treatment. Any man who wishes to see at his leisure a real variety of cases, will do well to leave the over-crowded London schools and devote a couple of years to a provincial hospital like the "Birmingham General." A large amount of good work is done here, but it may be doubted if the most is made of educational advantages. The committee do not appear to encourage the idea of making the place a "teaching hospital." Hence officials are burdened with much of the actual clerical work that in other similar hospitals with medical schools is done by students.

